# Assessment of the Effects of Anatoxin-a In Vitro: Cytotoxicity and Uptake

**DOI:** 10.3390/toxins16120541

**Published:** 2024-12-13

**Authors:** Cristina Plata-Calzado, Ana I. Prieto, Ana M. Cameán, Angeles Jos

**Affiliations:** Area of Toxicology, Faculty of Pharmacy, Universidad de Sevilla, Profesor García González 2, 41012 Seville, Spain; cpcalzado@us.es (C.P.-C.); camean@us.es (A.M.C.); angelesjos@us.es (A.J.)

**Keywords:** anatoxin-a, cytotoxicity, in vitro, uptake, gene expression, risk assessment

## Abstract

Anatoxin-a (ATX-a) is a cyanotoxin whose toxicological profile has been underinvestigated in comparison to other cyanotoxins such as microcystins (MCs) or cylindrospermopsin (CYN). However, its wide distribution, occurrence, and toxic episodes justify more attention. It is classified as a neurotoxin, but it has also been reported to affect other organs and systems. Thus, the aim of this study was to establish, as a first tier in its toxicological evaluation, its cytotoxicity in a wide range of cell lines representative of potential target organs (N2a, SH-SY5Y, HepG2, Caco2, L5178Y Tk^+/−^, THP-1 and Jurkat). As limited effects were observed after exposure to up to 200 µg/mL of ATX-a for 24 h (only Jurkat and THP-1 cells showed reduced cell viability), cell uptake experiments were performed by ultra-high performance liquid chromatography–tandem mass spectrometry (UHPLC-MS/MS). The results showed that the immune system cells had the highest percentage of ATX-a in the intracellular fraction, followed by neuronal cells and finally Caco-2 and HepG2 cells. Moreover, the expression of genes related to cell death mechanisms in THP-1 cells was also analyzed by polymerase chain reaction (PCR) and showed no changes under the conditions tested. Further research is required on ATX-a’s toxic effects and toxicokinetics to contribute to its risk assessment.

## 1. Introduction

Cyanobacterial blooms can pose a risk to the environment and public health as a result of their ability to produce cyanotoxins. Factors such as climate change and pollution from anthropogenic activity can lead to an increase in the frequency of such blooms. Therefore, the investigation of the toxic effects produced by cyanotoxins such as microcystins (MCs), cylindrospermopsin (CYN) or anatoxin-a (ATX-a) is a priority [1,2]. Of all these cyanotoxins, ATX-a is the least studied. According to the World Health Organization (WHO), more toxicological data are needed in order to update the provisional reference values established for ATX-a [3]. ATX-a is a secondary amine alkaloid produced by numerous cyanobacteria genders such as *Anabaena*, *Aphanizomenon*, *Oscillatoria*, or *Microcystis* [4].

ATX-a has been reported in Africa [5,6], Asia [7,8], Europe [9,10,11,12], America [13,14] and Oceania [15,16]. Furthermore, its presence is not limited to freshwater, but several studies have reported ATX-a in brackish water [17,18]. In terms of detected concentrations of ATX-a, this cyanotoxin typically remains below 10 µg/L in open water [3], although concentrations higher than 1000 µg/L have been documented in surface water [19]. Moreover, Wood et al. [20] detected up to 100-fold variations in the amount of ATX-a in toxic strains isolated from the same mat. These findings reveal that there is a large variability in the ATX-a content in different environmental samples, so toxic effects also need to be investigated in a wide concentration range.

Regarding its toxicity, it is important to note that several studies have described animal poisonings, mainly in dogs, due to exposure to this cyanotoxin [21,22,23,24,25]. In humans, the routes of exposure to ATX-a include inhalation, dermal, and oral routes, with the latter being the most relevant due to the consumption of contaminated water or food (mainly fish and vegetables) [26]. In this regard, human intoxication has recently been described as the result of the ingestion of ATX-a-contaminated sea figs [27], as well as the presence of ATX-a in bivalve samples at the Ingril site and at the Le Scoré site [28]. The results of these studies highlight the need to carry out ATX-a toxicity studies to clarify the consequences of the exposure to this cyanotoxin.

Early toxicity studies of ATX-a in isolated organs focused on demonstrating the mechanism of action, observing a high isomer-dependent affinity for nicotinic acetylcholine receptors (AChR) [29,30,31], with the (+)-ATX-a isomer being more than 100 times more potent than the (−)-ATX-a isomer [30]. Binding of ATX-a to AChR produces a neuromuscular blockade that results in symptoms such as fatigue, convulsions, paralysis, and respiratory failure, among others [3]. For this reason, ATX-a is classified mainly as a neurotoxin. Nevertheless, few studies have explored the toxic effects of this cyanotoxin using neuronal cell lines and have shown conflicting results. Thus, Takser et al. [32] studied the toxic effects of ATX-a on the N2a cell line. This study showed a significant reduction in N2a viability compared to the control group by MTT assay after exposure for 24, 48 and 72 h to 0.1 and 10 µM ATX-a. However, Kubickova et al. [33] did not observe ATX-a cytotoxicity in any of the assays performed (neutral red uptake, reduction in resazurin, lactate dehydrogenase release, acetoxymethyl ester cleavage and carboxyfluorescein diacetate) in human neural stem cells derived from the H9 cell line after exposure to concentrations of up to 20 µM ATX-a for 4 days.

In addition, interestingly, the effects of ATX-a on other tissues and systems have been reported. Thus, ATX-a has been shown to exhibit immunotoxic [32,34,35,36,37], cytotoxic [38,39], genotoxic [40] or cardiovascular effects [41,42,43]. However, most of these studies have been carried out on isolated organs or cells or used in vivo models.

Overall, although in vitro studies are one of the first steps in the toxicity evaluation of compounds, studies focused on the toxic effects of ATX-a in established cell lines are limited. Therefore, the aim of the present study was to investigate the potential cytotoxicity of ATX-a in different cell lines as the first tier in the process to elucidate its toxicological profile. Specifically, neuronal cell lines (N2a, SH-SY5Y), immune system cell lines (THP-1, Jurkat and L5178Y Tk^+/−^), and cell lines representative of an oral exposure (HepG2 and Caco-2) were employed, in which the possible cytotoxic effects of this cyanotoxin have been scarcely or not yet explored. Furthermore, in order to correctly interpret the results obtained in cell viability assays, the ATX-a uptake by the seven cell lines used was also analyzed, and for comparative purposes, CYN cytotoxicity and uptake in THP-1 cells were also included. Finally, the effects on the expression of genes related to apoptosis and necrosis mechanisms were explored in the cell line in which the highest cytotoxic effect of ATX-a was observed (THP-1 cell line).

## 2. Results

### 2.1. Cytotoxicity Assays

**Figure 1:**
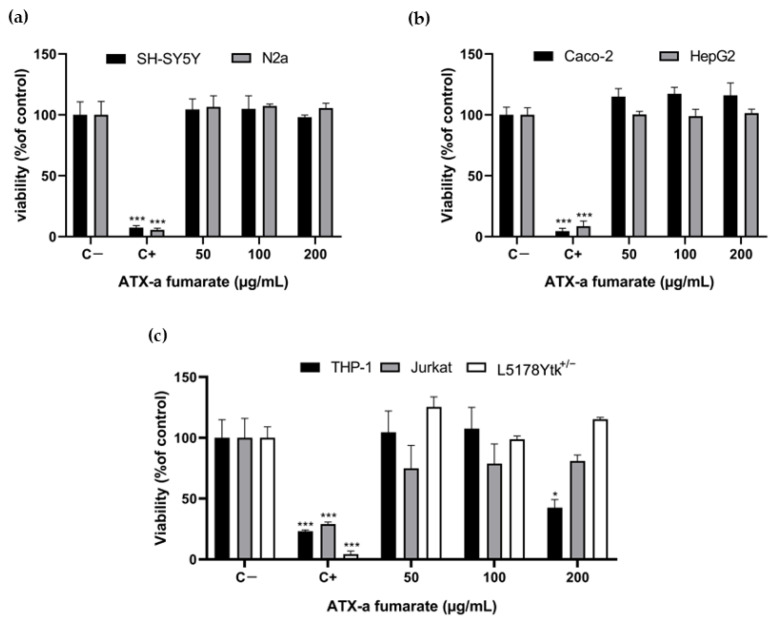
Reduction in tetrazolium salt MTS in (**a**) neuronal cells (SH-SY5Y and N2a), (**b**) Caco-2 and HepG2 cells and (**c**) immune system cells (THP-1, Jurkat and L5178Y Tk^+/−^) after 24 h of exposure to 0–200 µg/mL ATX-a fumarate. In addition, 0.3% *v*/*v* Triton X-100 was used as the positive control. All experiments were performed at least in triplicate per concentration. Data are expressed as mean ± SD compared to negative control group. Note: * *p* < 0.05 and *** *p* < 0.001 indicated significant difference from negative control.

The results obtained in the cytotoxicity assays with ATX-a are shown in Figure 1. A non-significant reduction in viability was observed in neuronal cells (SH-SY5Y and N2a) after 24 h of exposure to 0–200 µg/mL of ATX-a fumarate (equivalent to 0–117.5 µg/mL pure ATX-a) (Figure 1a). Similar results were obtained in the Caco-2 and HepG2 cell lines after the same exposure conditions (Figure 1b). A decrease in viability of the Jurkat cell line was observed after exposure to ATX-a fumarate for 24 h at all tested concentrations (Figure 1c). However, a significant decrease in cell viability was only obtained in the THP-1 cell line after exposure to the maximum concentration tested (200 µg/mL) compared to the control group.

**Figure 2:**
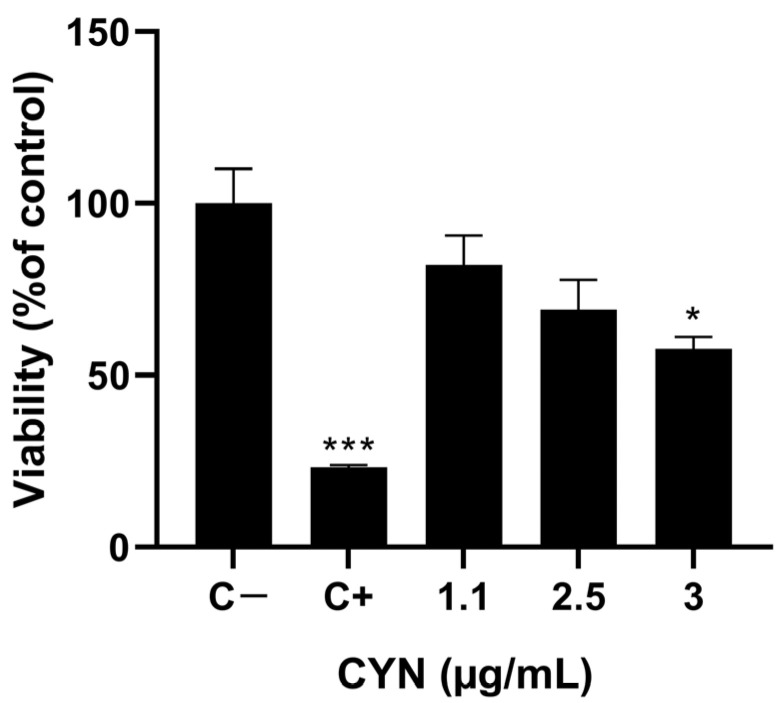
Reduction in tetrazolium salt MTS in THP-1 cells exposed to 0–3 µg/mL CYN for 24 h. In addition, 0.3% *v*/*v* Triton X-100 was used as the positive control. Experiments were performed in triplicate per concentration. Data are expressed as mean ± SD compared to negative control group. Note: * *p* < 0.05 and *** *p* < 0.001 indicate significant difference from the negative control.

As for CYN, a reduction in cell viability was observed at all concentrations employed. Specifically, an EC20 was obtained in THP-1 cells after exposure to 1.1 µg/mL of toxin for 24 h. The other concentrations assayed (2.5 and 3 µg/mL CYN) showed a viability of THP-1 cells 69.05 ± 8.7% and 57.67 ± 3.5%, respectively, compared to the negative control (Figure 2).

### 2.2. Uptake of ATX-a

**Figure 3:**
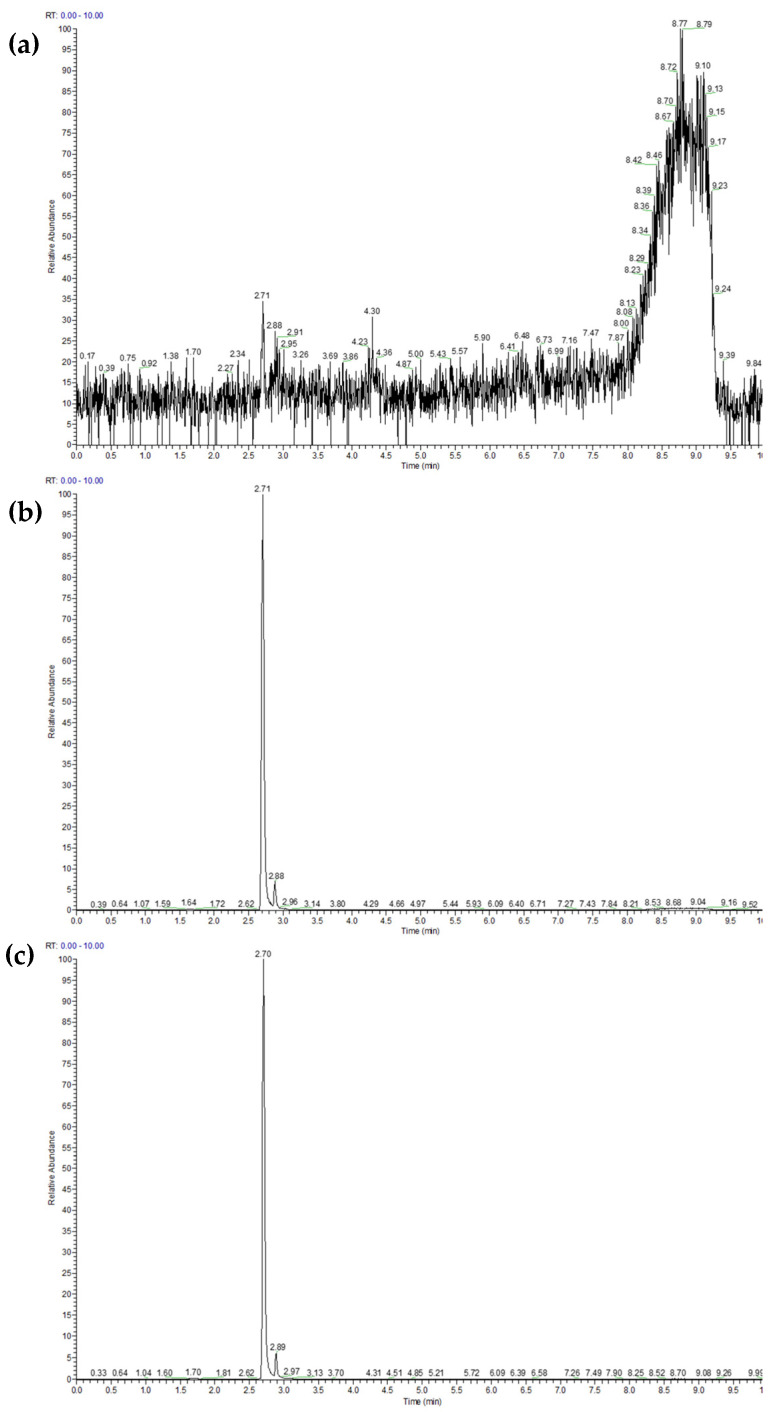
Chromatograms obtained by UHPLC-MS/MS of ATX-a in THP-1 cells exposed to 50 µg/mL ATX-a fumarate for 24 h. (**a**) Negative control, (**b**) intracellular fraction and (**c**) extracellular fraction.

An analysis of ATX-a uptake by the cell lines used in the cytotoxicity assays was performed by UHPLC-MS/MS. The limits of detection (LOD) and quantification (LOQ) of the analytical method were 1.88 and 6.28 µg/L, respectively. As an example, ATX-a uptake chromatograms in the THP-1 cell line after exposure to 50 µg/mL ATX-a fumarate for 24 h are shown (Figure 3). Chromatograms corresponding to the other six cell lines exposed to the same concentration of ATX-a fumarate are available in the Supplementary Material (Figures S1–S3).

The results obtained in the determination of ATX-a in the cell lines assayed are shown in Table 1. In this way, taking into account the amount of toxin added, between 43.55 and 129.84% of ATX-a was detected in the analysis. The results showed ATX-a in the intracellular fraction of all cells analyzed. However, the amounts detected in each of the cell types were different. Specifically, a higher uptake was observed in the following cell lines belonging to the immune system: L5178Y Tk^+/−^ (6.45–9.70%), Jurkat (10.58–13.96%) and THP-1 (6.62–14.72%) of total ATX-a added, followed by neuronal cell lines (between 1.52 and 2.94%) and finally, in Caco-2 and HepG2 (between 0.30 and 0.65% of total toxin added) (Table 1).

### 2.3. Uptake CYN

The results of CYN uptake in the THP-1 cell line expressed as % of CYN detected with respect to the total amount of toxin added are shown in Table 2. The percentages of detected CYN ranged from 57.81 to 65.85%. An increase in the percentage of intracellular CYN was observed as the concentration of exposure to this cyanotoxin increased.

### 2.4. Gene Expression Analysis by Quantitative Real-Time PCR

**Figure 4:**
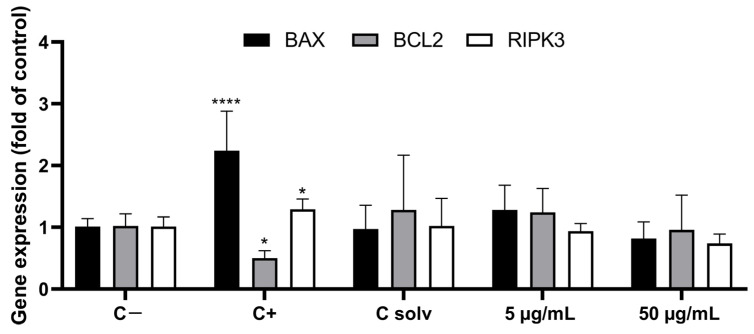
Effects of ATX-a on the expression of mRNA of genes involved in apoptosis/necrosis in THP-1 cells. Cells were exposed to 5 or 50 µg/mL ATX-a fumarate for 24 h. CPT (0.5 µM) was used as positive control and chloroform/methanol (4:1) as solvent control. Results are expressed as relative mRNA expression normalized to the negative control group. * *p* < 0.05 and **** *p* < 0.0001 indicate significantly difference from negative controls.

The effects of ATX-a exposure for 24 h on mRNA expression of genes involved in cell death mechanisms were analyzed in the THP-1 cell line by RT-qPCR (Figure 4). ATX-a did not produce changes in the expression of the genes investigated at either of the two concentrations assayed (5 and 50 µg/mL ATX-a fumarate) under the conditions tested. Changes in gene expression were only observed after exposure to the positive control: upregulation in BAX and downregulation in BCL2.

## 3. Discussion

Although ATX-a is a well-known cyanotoxin, the amount of studies and experimental data dealing with it is lower compared to other cyanotoxins such as microcystins [44] or cylindrospermopsin [45], as it was evidenced by the review performed by Plata-Calzado et al. [46]. However, it is a widely reported toxin in important cases of deaths of dogs, livestock and wild animals poisoning, with convulsions, ataxia and paresthesia of cerebral hypoxia being some of the main toxic effects observed after ATX-a exposure [3]. In addition, the WHO pointed out that the toxicological database on ATXs was not adequate to support derivation of a formal guideline value [3]. Thus, to further explore the mechanisms of ATX-a toxicity, cell viability studies with different established cell lines were performed. These assays are a basic approach in toxicity-testing strategies and could support the establishment of the adverse outcome pathways (AOPs) of the studied chemical. As ATX-a is primarily classified as a neurotoxin, two different neuronal cells, SHSY-5Y and N2a, were first selected. Both have been reported to express AChR, of which ATX-a is an agonist [47,48]. Surprisingly, no evident cytotoxicity was observed at low concentrations on SH-SY5Y, but even at high concentrations (up to 200 µg/mL) in both cell lines. On the contrary, CYN, although not specifically classified as a neurotoxin, was reported to induce a higher cytotoxicity on SHSY-5Y cultures, with an EC_50_-24h value of only 0.87 ± 0.13 µg/mL using the same assay [49]. The number of studies of ATX-a in neuronal stablished cell lines is limited. Only Takser et al. [32] reported cytotoxicity in N2a cells after exposure to 0.1 and 10 µM ATX-a (equivalent to ≈0.028 and 2.8 µg/mL, respectively) in the MTT assay, although the effects were of similar magnitude, without a concentration-dependent pattern. In addition, an EC_50_ was only reached after 72 h of exposure to ATX-a. In contrast, these ATX-a concentrations did not induce cytotoxicity in BV-2 cells (murine microglial cells).

As cyanotoxins are reported to induce toxicity at different levels, other established cell lines of additional target organs were selected to test ATX-a in order to check cytotoxicity responses. Thus, HepG2 and Caco-2 cells were used taking into account that oral exposure is the main exposure for cyanotoxins in the general population. But results provided a similar outcome, no evident cytotoxicity. To our knowledge, no studies on the possible in vitro adverse effects of ATX-a on the gastrointestinal system have been published, although alterations in enzyme biomarkers have been detected in fish liver after intraperitoneal administration of ATX-a [50].

Additionally, cells lines related to the immune system (monocytes and lymphoblasts) were selected. In this case, L5178Y Tk^+/−^ cells did not experience toxic effects, Jurkat cells reduced its viability in a non-significant way, and THP-1 cells significantly reduced its viability at the highest concentration tested (200 µg/mL). Scientific literature reports toxic effects of ATX-a on the immune system. Thus, Takser et al. [32] observed cytotoxicity in the RAW246.7 murine macrophage-like cell line exposed to 0.1 and 10 µM ATX-a. Moreover, ATX-a has shown its toxicity on immunological models from fish [36,37] but using isolated cells and not established cell lines, making the comparison more difficult. Regarding mammalian models, apart from Takser et al. [32], the most relevant studies to compare with would be those by Rao et al. [34] and Teneva et al. [35]. Rao et al. [34] exposed thymocytes isolated from 4- to 8-week-old rats to (+)-ATX-a and observed a strong concentration-dependent decrease in cell viability with a total reduction at 50 µg/mL. Teneva et al. [35] also observed a significant cytotoxicity in spleen cells isolated from male 8-week-old BALB/c mice exposed to 0.1 µg/mL for 24 h, with no specification of the isomer used. These differences observed with respect to our study could be due to the ATX-a isomer used. Thus, Zhang et al. [30] found that (+)-ATX-a was 160-fold more potent than (−)-ATX-a, and 20-fold more potent than (±)-ATX-a in inhibiting [^3^H]ACh binding in rat brains. However, Molloy et al. [31] used synthetic (±)-ATX-a (up to 100 µg/mL) in bovine adrenal chromaffin cells showing that ATX-a was a potent nicotinic acetylcholine receptor agonist in these cells. Similarly, Liu et al. [51] also used the racemic mixture, and they reported agonist activity in the yeast oestrogen screen assay in *Saccharomyces cerevisiae*. In our study, the concentrations of (+)-ATX-a contained in the racemic mixture exceed those used by Rao et al. [34] and Teneva et al. [35], so the experimental model (primary culture versus established cell line) could be the main reason for the differences found with respect to our results.

Thus, considering the cytotoxicity data obtained from a general point of view, it has been evidenced that ATX-a showed a limited cytotoxicity in the established cell lines tested, and that the type of experimental model used could play a role. However, Takser et al. [32] used the same cell line (N2a), a similar assay (MTT assay, based also on the mitochondrial enzyme succinate dehydrogenase activity), and the same toxin (ATX-a from Enzo) and they observed cytotoxicity. More recently, Kubickova et al. [33] have tested ATX-a using a differentiating in vitro human neural stem cell model and observed that ATX-a (up to 20 µM) did not cause distinct developmental neurotoxicity alone, and that its (excito-)toxicity to acetylcholinergic neurons occurred only in co-exposure to all-trans retinoic acid.

The results could also be explained due to the non-effective uptake of ATX-a by the cells. Thus, other widely studied cyanotoxins, such as MCs [2], are known to require specific transporters to enter the cells. They are uptaken by the organic acid transporter polypeptides (OATPs) and limited toxicity on established cell lines has been explained by the lack of these specific transporters [52]. Unfortunately, data regarding ATX-a kinetics are limited, and no reports suggest the participation of transporters. Thus, Testai [2] stated that ATX-a is passively and rapidly absorbed after ingestion and widely disseminated to different tissues, including the brain. Thus, to check the uptake of ATX-a by the cells, the determination of the toxin was performed in the supernatants and inside the cells after 24 h of exposure to the toxin. The results showed that the uptake in all cell lines was limited. Cells of immunological nature showed a higher intracellular presence than those from the nervous system and from the gastrointestinal system. This was in agreement with the cytotoxicity results. Also, an interesting observation derived from the data obtained is that the uptake (intracellular recovery) was concentration-dependent in all cell lines, with the exception of the THP-1 and HepG2 cell lines, where the amount of toxin detected in the intracellular fraction was similar at both concentrations. Therefore, at least a small part of ATX-a could be taken up by passive diffusion, which would explain why it is detected inside all cell lines and that the amount was concentration-dependent. In addition, the main aim of the present work was to analyze whether the content of uptaken toxin was relevant to induce cytotoxicity. However, the results do not seem to show a relationship between uptake and the observed toxic effects, and with that aim, a similar experiment with CYN was performed for comparative purposes. Thus, cytotoxicity-causing CYN concentrations were used, and the percentage of cytotoxicity and uptake was analyzed. The cytotoxicity assays performed by Casas-Rodriguez et al. [53] were replicated in the THP-1 cell line, obtaining similar results (EC_20_ = 1.1 µg/mL and approximately EC_50_ at a concentration of 3 µg/mL CYN). Regarding toxin uptake, a concentration-dependent increase in intracellular % was also observed. However, the % intracellular recovery was very low and even much lower than in the case of ATX-a, and despite this, an evident cytotoxicity was observed. Therefore, the absence of ATX-a cytotoxicity is not only a consequence of low toxin uptake.

A different hypothesis to try to explain the absence of effects is that cytotoxicity is a non-very sensitive biomarker of cell damage, and very high concentrations are required. This argument was used by Menezes et al. [54] for the toxic effects induced by MC-LR in the kidney Vero-E6 cell line. They concluded that the type and extent of effects were largely influenced by the toxin concentration, noting that certain responses, like cell death by apoptosis or necrosis, need much higher concentration compared to others, such as the expression of mitogen-activated protein kinase (MAPK). In order to test this hypothesis, the expression of genes involved in cell death was analyzed in the cell line most sensitive to cytotoxic effects, THP-1 cells. Also, it is known that for other cyanotoxins, such as CYN, low concentrations (EC_20_ values) can already induce important changes at a genetic level [53]. However, in this case, again, no effects were observed. Nevertheless, we cannot dismiss the possibility that the lack of direct cytotoxicity of ATX-a could imply a potential ATX-a toxicity mediated by other responses such as interactions with certain molecular receptors or altering cellular processes that do not result in immediate cell death. On the other hand, in comparison to CYN, it might happen that the limited in vitro cytotoxicity observed for ATX-a is just a matter of toxic potency. Thus, CYN has proved to be more cytotoxic than ATX-a on the different cell lines studied [49,53,55,56]. Regarding in vivo data, median lethal dose values after acute exposure reported by the WHO [3,57] were lower for CYN than for ATX-a, but a direct comparison is not possible due to differences in exposure, purity and concentrations of toxin used, etc.

Overall, the results obtained in this research reveal important issues regarding the selection of the most suitable experimental models and biomarkers to use in ATX-a toxicological research. However, the observation that ATX-a is taken up by all cell lines tested suggests that this toxin can potentially affect various tissue types and that there is no cytotoxic potential in these cell models under the conditions tested. In view of the results obtained, further research with more advanced and sensitive experimental models is needed to elucidate their possible absorption pathways and toxicity mechanisms, in order to clarify their toxic potential to support an adequate risk assessment.

## 4. Conclusions

The cytotoxicity of ATX-a in the cell lines evaluated was low even at high concentrations (200 µg/mL). This may be due to different reasons among which the experimental model, isomer specificity of toxin, toxin uptake or the cell damage marker used stand out. The ATX-a uptake results showed toxin inside all the cell lines studied, concluding that the absence of cytotoxicity was not due to a limited uptake. In contrast, CYN with a lower uptake induced higher cytotoxicity. Also, no response was observed in the expression of genes related to the apoptosis and necrosis response. The results seem to indicate that the established cell lines might not be a suitable model to study ATX-a toxicity, and that ATX-a toxic potency is lower than for CYN. Thus, although the established cell lines are a first step in the investigation of the toxic effects of substances, the present study shows that in the case of ATX-a, common cell models such as N2a, SH-SY5Y, THP-1, Jurkat, L5178Y Tk^+/−^, HepG2 and Caco-2 cell lines were not the most appropriate. Studies with more advanced models such as primary cell cultures or in vivo systems are needed to better understand the mechanisms and toxic effects of ATX-a.

## 5. Materials and Methods

### 5.1. Chemicals

(±) Anatoxin-a fumarate standard with a purity greater than 98.0% and cylindrospermopsin with a purity of 95% were provided by Enzo Life Sciences (Lausen, Switzerland). The reagents needed for cell culture were sourced from Gibco (Biomol, Sevilla, Spain). Reagents for cyanotoxin extraction and LC-MS/MS analysis were obtained from Sigma–Aldrich (Madrid, Spain). Finally, the chemical reagents used for RT-qPCR were provided by Qiagen (Madrid, Spain) and Bio-Rad Laboratories (Hercules, CA, USA).

### 5.2. Cell Lines and Culture Conditions

The cell lines as well as the culture conditions used are detailed in Table 3. The recommendations of the supplier of the cell lines (the American Type Culture Collection (ATCC)) for their maintenance were followed.

### 5.3. Test Solutions

Stock solutions of ATX-a fumarate (1000 µg/mL) and CYN (1000 µg/mL) were prepared in milli-Q sterile water and maintained at −20 °C. Toxin concentrations were prepared in the corresponding cell culture medium.

### 5.4. Cytotoxicity Assays

For cytotoxicity assays, cells were seeded at different densities depending on the growth of each cell line in 96-well culture plates and following the supplier’s recommendations. Thus, N2a, SH-SY5Y and L5178Y Tk^+/−^ were seeded at 2 × 10^5^ cells/mL, THP-1 and HepG2 at 3 × 10^5^ cells/mL, Jurkat at 5 × 10^5^ cells/mL and Caco-2 at 7.5 × 10^5^ cells/mL. Cells were incubated at 37 °C and 5% CO_2_ for 24 h until 70% confluence was reached. Subsequently, cells were exposed to concentrations of ATX-a fumarate ranging from 0 to 200 µg/mL (equivalent to 0–117.5 µg/mL pure ATX-a). Previously, preliminary cytotoxicity assays were performed on the SH-SY5Y cell line with lower concentrations of ATX-a fumarate (2.5, 5, 7.5, 10, 15, 20, 40, and 60 µg/mL) for 24 h. No decrease in cell viability was observed compared to the control group at those concentrations, so higher concentrations were considered for further cytotoxicity assays (up to 200 µg/mL ATX-a fumarate). The limit of 200 µg/mL was established taking into account the data reported on the highest ATX-a concentrations found in nature (1000 µg/L) [19]. To check appropriate test conditions, Triton X-100 (0.3% *v*/*v*) with a recognized cytotoxic effect was introduced into the assay as a positive control. The cells were treated with the corresponding concentration for 24 h. The biomarker assayed was the reduction in the tetrazolium salt MTS (3-(4,5-dimethylthiazol-2-yl)5-(3-carbox-ymethoxyphenyl)-2-(4-sulfophenyl)2H-tetrazolium salt) assay following the instructions of the manufacturer’s protocol for the CellTiter 96^®^ Aqueous One Solution Cell Proliferation Assay (Promega, Madrid, Spain). After 2 h of incubation with MTS, the absorbance was measured at 492 nm by absorption spectrophotometry. Cell viability obtained at the exposure concentrations was expressed as % with respect to the negative control.

As ATX-a did not reduce cell viability to a high extent, a parallel cytotoxicity assay with CYN was performed in THP-1 cells following the same protocol to compare the cytotoxicity produced with the uptake of both toxins by this cell line, since they both are hydrophilic cyanotoxins that can be found in nature simultaneously [22]. Therefore, cells were exposed to concentrations of CYN ranging from 0 to 3 µg/mL to obtain effective concentration values (EC_20_ and EC_50_), according to previously published results [53].

### 5.5. Evaluation of ATX-a Uptake

#### 5.5.1. Exposure

The cell treatment was the same as described above for cytotoxicity assays (Section 2.4) but with ATX-a fumarate concentrations of 50 and 100 µg/mL (equivalent to 29.4 and 58.7 µg/mL ATX-a). After exposure time, cells and medium were collected separately and stored at −20 °C until further analysis. For adherent cells, after collecting the medium, 0.05% (1X) trypsin-EDTA was added to lift the cells and stored. For suspension cells, cells were centrifuged 5 min at 2000× *g* rpm, the supernatant was collected, and the cells were resuspended in 50 µL of fresh medium and stored at −20 °C. All exposure treatments were performed by triplicate.

#### 5.5.2. Extraction and Purification Procedures

The ATX-a content was extracted following the protocol of Dimitrakopoulus et al. [58]. To extract the toxin, 5 mL of 20% methanol (MeOH) was added to the cell samples and the supernatants and sonicated for 10 min. Then, samples were centrifuged at 3700× *g* rpm for 30 min. The supernatant was collected, and the pH was adjusted to 10.5 with NaOH. Briefly, graphitized carbon cartridges (Bond Elut cartridges) supplied by Agilent Technologies (Santa Clara, CA, USA) were used for solid-phase extraction (SPE). The SPE cartridge was preconditioned with 6 mL of MeOH and equilibrated with 6 mL of water. Subsequently, the sample at pH 10.5 was passed through the cartridges and eluted with 6 mL of MeOH + 0.1% trifluoroacetic acid. The resulting extracts were evaporated to dryness in a rotary evaporator and resuspended in 1 mL MeOH 20% prior to analysis by ultra-high performance liquid chromatography–tandem mass spectrometry (UHPLC-MS/MS).

#### 5.5.3. UHPLC-MS/MS

In order to carry out the determination of ATX-a, the possible interferences that can be caused by the amino acid phenylalanine have been taken into account [59]. Thus, previous analyses were performed with a standard of ATX-a and phenylalanine, with the aim of verifying that the chromatographic conditions allowed the correct differentiation of both compounds. Therefore, ATX-a determination was carried out using a Thermo Scientific liquid chromatography system consisting of a binary UHPLC Dionex Ultimate 3000 RS, connected to a quadrupole-orbitrap QExactive hybrid mass spectrometer (ThermoFisher Scientific, Waltham, MA, USA) with a HESI ionization probe. UHPLC analyses were performed on a 100 × 2.1 mm Acquity HSS T3 1.8 µm column. The flow rate was 0.450 mL/min. A gradient of (A) water and (B) acetonitrile, each containing formic acid (FA) (*v*/*v*) in a proportion of 0.1%, was achieved. An injection volume of 5 µL was also set. The profile of elution was as follows: 2% B for 0.8 min, then 70% B for 9 min, 80% B for 1 min, and the last 2 min with 2% B. A Parallel Reaction Monitoring (PRM) method was employed in positive mode. The HESI source parameters were sheath and auxiliary gas flow rates, 60 and 25 arbitrary units, respectively; spray voltage, 3.5 kV; capillary temperature, 320 °C; S-lens RF level, 50; and probe heater temperature, 430 °C. The PRM method was used at a resolution of 70,000 at *m*/*z* 200 full width at half maximum (FWHM), and the monitored pseudomolecular ion [M+H]+ had a *m*/*z* of 166.12264.

In addition, ATX-a uptake calculations took into account the amount of toxin present in the reference standard used (ATX-a fumarate), according to the following relation: *Corrected mass = (MW_anatoxin-a_/MW_anatoxin-a fumarate_) × Measured mass.*

### 5.6. Evaluation of CYN Uptake

#### 5.6.1. Exposure, Extraction and Purification Procedures

Treatment of THP-1 cells with CYN concentrations of 1.1, 2.5 and 3 µg/mL was performed as described above for cytotoxicity assays (Section 5.4). After 24 h of exposure, cells were centrifuged 5 min at 2000× *g* rpm, the supernatant was collected, and the cells were resuspended in 50 µL of fresh medium and stored at −20 °C, following the same methodology as for ATX-a uptake assessment.

After that, CYN was extracted using the protocol validated by Guzmán-Guillén et al. [60]. Firstly, to extract the toxin, 3 mL of milliQ water was added to the cell samples and the supernatants and sonicated for 15 min. Then, samples were stirred for 1 h and sonicated 15 min more. Subsequently, the samples were centrifuged at 3700× *g* rpm for 10 min. In addition, 6 µL trifluoroacetic acid was added to the supernatant. Finally, it was shaken for 1 h and stood for 3 h.

Regarding clean-up, graphitized carbon cartridges (Bond Elut cartridges) were used for SPE. The SPE cartridge was preconditioned with 10 mL of DCM/MeOH (10/90) with 5% FA and then equilibrated with 10 mL of milliQ water. Subsequently, the sample was passed through the cartridges, washed with 10 mL of milliQ water and eluted with 10 mL of the acidified DCM/MeOH (10/90) solution. The resulting extracts were evaporated to dryness using a rotary evaporator and resuspended in 500 µL milliQ water before analysis by UPLC-MS/MS analysis.

#### 5.6.2. UPLC-MS/MS Determination of CYN

An UPLC Acquity instrument (Waters, Milford, MA, USA) was used to perform the chromatographic separation. This equipment was connected to a Xevo TQS-micro instrument (Waters, Milford, MA, USA), which consists of a triple quadrupole mass spectrometer, and utilized an electrospray ionization source operation in positive mode. The separation was conducted on an Acquity BEH C18 column (50 × 2.1 mm, 1.7 µm particle size) with a flow rate of 0.45 mL/min. The mobile phase consisted of (A) water containing 0.1% FA (*v*/*v*) and (B) methanol with 0.1% FA (*v*/*v*). The gradient elution profile was programmed as follows: 0% B for 0.8 min, followed by a linear increase to 90% B over 2.2 min, maintained at 90% B for 1 min, and finally returned to 0% B for 1 min. The injection volume was set at 5 µL. For detection, Multiple Reaction Monitoring (MRM) was employed, with parent ions monitored in Q1 and fragments ions in Q3. For quantification, the transition of CYN used was 416.2/194.0 and for confirmation, it was 416.2/176.0. The mass spectrometer parameters were optimized as follows: capillary voltage at 3.0 kV, desolvation gas flow at 1000 L/h, source desolvation temperature at 500 °C, and cone gas flow at 50 L/h.

### 5.7. Real-Time Quantitative PCR (qRT-PCR) Analysis After ATX-a Exposure

For gene expression analysis, THP-1 cells were selected because they showed a higher cytotoxicity of the toxin. Cells were seeded at 5 × 105 cells/mL and incubated for 24 h at 37 °C in 5% CO_2_. Subsequently, cells were exposed to fresh medium containing 5 and 50 µg/mL ATX-a fumarate and incubated for 24 h. The concentrations were selected according to the concentrations found in nature (equivalent to 5 µg/mL ATX-a fumarate) [61,62] and for comparison against the results obtained in the rest of tests (50 µg/mL ATX-a fumarate). Although these concentrations did not decrease cell viability, alteration of gene expression is a molecular event induced at lower concentrations than cytotoxicity [52,54].

Cell medium was used as a negative control and camptothecin (CPT) (Sigma-Aldrich, C9911, Madrid, Spain) at 0.5 µM dissolved in chloroform/methanol (4:1) was used as a positive control. For this reason, a solvent positive control (chloroform/methanol 4:1) was introduced in the assay. After ATX-a treatment, cells were centrifuged at 300× *g* for 5 min and resuspended with 1 mL of fresh medium. RNA was extracted according to the manufacturer’s instructions for the RNAeasy Mini Kit (Cat: 74104, Qiagen, Madrid, Spain). In addition, RNA purification was carried out using the RNAse-free DNAse set (Cat: 79254). After that, RNA purity was measured using the ratio 260/280 nm and 260/230 nm in NanoDrop 2000 (Thermo Scientific, Pittsbutgh, PA, USA). The samples were stored at −80 °C until reverse transcription was performed. In addition, 1 µg of total RNA was used to obtain cDNA by reverse transcription (RT) using the QuantiTec^®^ reverse transcription kit (Cat: 205311, Qiagen, Madrid, Spain) according to the supplier’s instructions. Samples were kept at −20 °C until quantitative real-time polymerase chain reaction assay (RT-qPCR) was performed. The expression of apoptosis/necrosis genes including B-cell lymphoma 2 (BCL2), Bcl-2-associated X protein (BAX) and receptor-interacting Serine/Threonine-Protein Kinase 3 (RIPK3) were analyzed. Also, the glyceraldehyde-3-phosphate dehydrogenase (GAPDH) housekeeping gene was used for the calculations. The primers used are shown in Table S1. A 1:5 dilution of cDNA in RNAse-free water was performed and amplified by PCR in a 384-well plate. Diluted cDNA, the prime PCR probe (Bio-Rad Laboratories, Inc., Hercules, CA, USA) for the corresponding gene and the iTaq universal probes Supermix (Cat: 1725134) were used for the reaction in a final volume of 10 µL. Amplification was performed using the Light Cycler^®^480 System (Roche, Berlin, Germany) with an initial denaturation step at 95 °C for 2 min followed by 50 cycles of 95 °C for 5 seg and 60 °C for 30 seg. The results were determined using the 2^−ΔΔCT^ method.

### 5.8. Statistical Analysis

The distribution of the data was checked with the Kolmogorov–Smirnov test. For statistical analysis, data with a normal distribution were analyzed using the analysis of variance (ANOVA) followed by Dunnett’s multiple comparison. For data that did not follow a normal distribution, the Kruskal–Wallis test was employed, followed by Dunn’s multiple comparison tests. All results were analyzed with Graph-Pad Prism 8.0.1 Software (Graph-Pad Prism 8 Software Inc., La Jolla, CA, USA). The results were expressed as mean ± standard deviation (SD). Differences were considered significant at * *p* < 0.05, ** *p* < 0.01, *** *p* < 0.001 and **** *p* < 0.0001, respectively.

## Figures and Tables

**Table 1:** Mean ± SD (%) of intracellular and extracellular ATX-a in N2a, SH-SY5Y, L5178YTk^+/−^, Jurkat, THP-1, HepG2, and Caco-2 cell lines after exposure to 50 or 100 µg/mL ATX-a fumarate for a period of 24 h by triplicate. n.d.: not detected.

	ATX-a Fumarate (µg/mL)	% Intracellular Mean ± SD	% Extracellular Mean ± SD	% Total ATX-a Detected Mean ± SD
N2a	C−	n.d.	n.d.	n.d.
	50 µg/mL	1.71 ± 1.15	59.90 ± 4.32	61.61 ± 3.59
	100 µg/mL	1.52 ± 0.66	42.03 ± 11.73	43.55 ± 11.36
SH-SY5Y	C−	n.d.	n.d.	n.d.
	50 µg/mL	1.99 ± 0.67	127.85 ± 8.71	129.84 ± 9.38
	100 µg/mL	2.94 ± 1.91	106.91 ± 39.49	109.85 ± 40.62
L5178YTk^+/−^	C−	n.d.	n.d.	n.d.
	50 µg/mL	9.70 ± 4.78	72.61 ± 9.54	82.31 ± 10.34
	100 µg/mL	6.45 ± 1.31	69.44 ± 10.50	75.89 ± 9.45
Jurkat	C−	n.d.	n.d.	n.d.
	50 µg/mL	10.58 ± 0.74	75.21 ± 23.23	85.78 ± 23.85
	100 µg/mL	13.96 ± 3.48	74.14 ± 15.58	88.11 ± 15.99
THP-1	C−	n.d.	n.d.	n.d.
	50 µg/mL	14.72 ± 4.55	93.90 ± 3.35	108.62 ± 7.46
	100 µg/mL	6.62 ± 1.37	56.35 ± 5.35	62.98 ± 4.80
HepG2	C−	n.d.	n.d.	n.d.
	50 µg/mL	0.65 ± 0.05	104.03 ± 2.82	104.72 ± 2.87
	100 µg/mL	0.30 ± 0.02	62.52 ± 4.87	62.81 ± 4.89
Caco-2	C−	n.d.	n.d.	n.d.
	50 µg/mL	0.46 ± 0.15	83.72 ± 30.85	84.18 ± 30.75
	100 µg/mL	0.52 ± 0.22	72.02 ± 21.88	72.54 ± 21.88

**Table 2:** Mean ± SD (%) of intracellular and extracellular CYN in THP-1 cells after exposure to 0. 1.1, 2.5 and 3 µg/mL CYN for a period of 24 h by triplicate. n.d.: not detected.

	CYN (µg/mL)	% Intracellular Mean ± SD	% Extracellular Mean ± SD	% Total CYN Detected Mean ± SD
THP-1	C−	n.d.	n.d.	n.d.
	1.1 µg/mL	0.38 ± 0.05	59.74 ± 1.01	60.12 ± 1.06
	2.5 µg/mL	0.71 ± 0.16	57.10 ± 2.19	57.81 ± 2.21
	3 µg/mL	1.30 ± 0.58	64.56 ± 4.29	65.85 ± 4.83

**Table 3:** Cell lines employed in the study and culture conditions for the maintenance.

Cell Line	Cell Type	Tissue Type	Model Organism	Culture Media	Culture Conditions
N2a	Neuroblast	Brain	Mouse	43.5% OptiMEM + 43.5% DMEM high glucose + 10% FBS, 1% piruvate 1% L-glutamine and 1% penicillin–streptomycin	Incubator at 37 °C with 95% relative humidity and 5% CO_2_
SH-SY5Y	Neuroblast	Brain	Human	43% MEM + 43% Ham’s F12 + 10% FBS, 1% piruvate 1% L-glutamine, 1% NEAA and 1% penicillin–streptomycin	
Jurkat	T lymphoblast	Peripheral blood	Human	RPMI 1640 medium containing high glucose (R8005, Sigma Aldrich) + 10% FBS, 1% penicillin–streptomycin and 2g/L sodium bicarbonate	
THP-1	Monocyte	Peripheral blood	Human		
L5178Y Tk^+/−^	Lymphoblast	Lymph node	Mouse	RPMI 1640 medium + 10% horse serum, 1% pyruvate, 1% L-glutamine and 1% penicillin–streptomycin	
Caco-2	Epithelial cell	Colon	Human	MEM medium + 10% FBS, 1% NEAA, 1% pyruvate, 1% L-glutamine and 1% penicillin–streptomycin	
HepG2	Hepatocyte	Liver	Human	MEM medium + 10% FBS, 1% L-glutamine and 1% penicillin–streptomycin	

DMEM: Dulbecco’s modified eagle; MEM: minimum essential medium FBS: fetal bovine serum; RPMI: Roswell Park Memorial Institute; NEAA: non-essential amino acids.

## Data Availability

The original contributions presented in this study are included in the article/Supplementary Material. Further inquiries can be directed to the corresponding author.

## Supplementary Materials

The following supporting information can be downloaded at https://www.mdpi.com/article/10.3390/toxins16120541/s1: Figure S1: Chromatograms obtained by UHPLC-MS/MS of ATX-a in L5178YTk^+/−^ and Jurkat cells exposed to 50 µg/mL ATX-a fumarate for 24 h. (a) Negative control, (b) intracellular fraction; (c) extracellular fraction. Figure S2: Chromatograms obtained by UHPLC-MS/MS of ATX-a in Caco-2 and HepG2 cells exposed to 50 µg/mL ATX-a fumarate for 24 h. (a) Negative control, (b) intracellular fraction; (c) extracellular fraction. Figure S3: Chromatograms obtained by UHPLC-MS/MS of ATX-a in N2a and SH-SY5Y cells exposed to 50 µg/mL ATX-a fumarate for 24 h. (a) Negative control, (b) intracellular fraction; (c) extracellular fraction. Table S1: Primers used for PCR reactions in this study for responses to apoptosis (BAX, BCL-2), and necrosis (RIPK3) and the housekeeping gene (GAPDH).
