# Supplementary material for: Assessment of the Effects of Anatoxin-a In Vitro: Cytotoxicity and Uptake
Source: Toxins (Basel). 2024 Dec 13;16(12):541. doi: 10.3390/toxins16120541 (PMC11680428; doi:10.3390/toxins16120541)
Supplement: Supplementary file 1 [file toxins-16-00541-s001.zip › toxins-3306171-supplementary.pdf]

# Supplementary Materials: Assessment of the Effects of Anatoxin-a In Vitro: Cytotoxicity and Uptake

**Cristina Plata-Calzado, Ana I. Prieto, Ana M. Cameán and Angeles Jos**

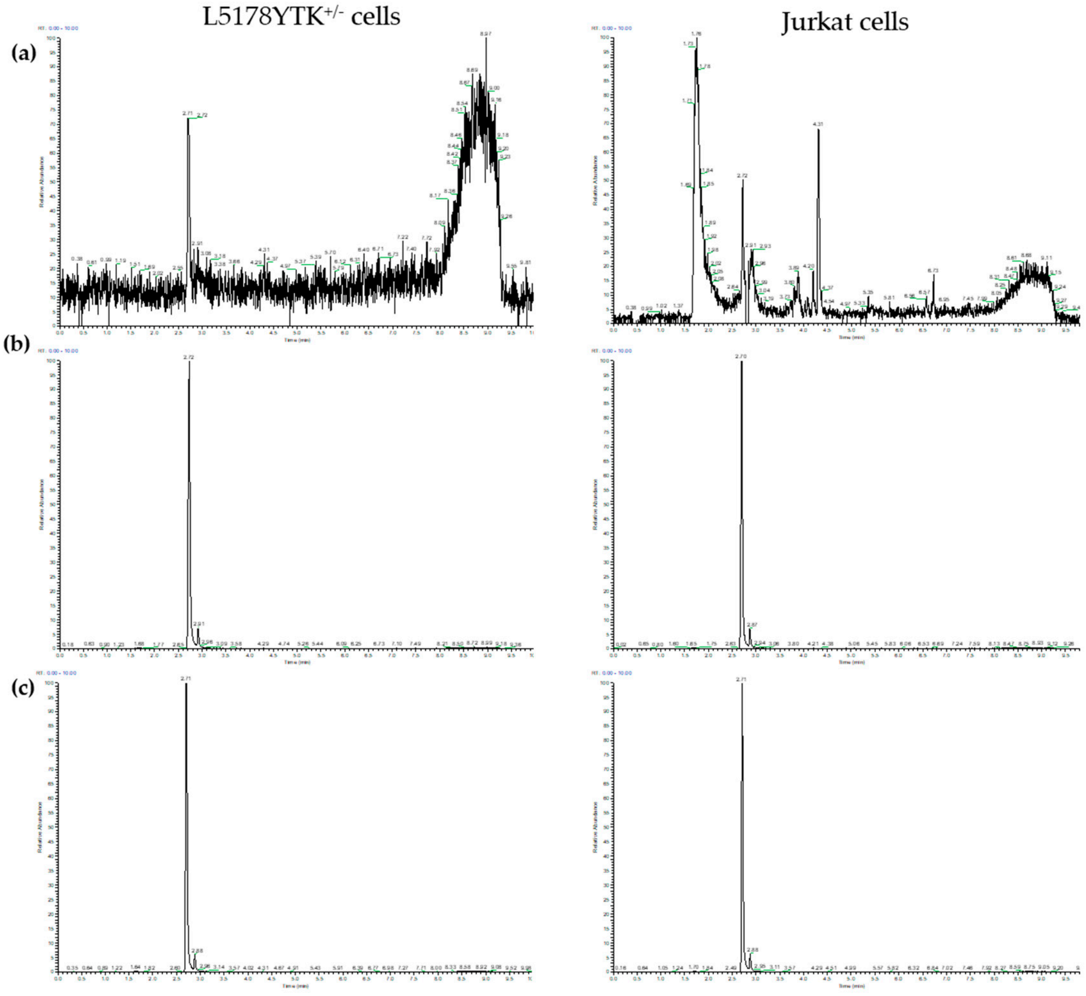


**Figure S1.** Chromatograms obtained by UHPLC-MS/MS of ATX-a in L5178YTk+/- and Jurkat cells exposed to 50 μg/mL ATX-a fumarate for 24 h. (a) Negative control, (b) intracellular fraction (c) extracellular fraction.

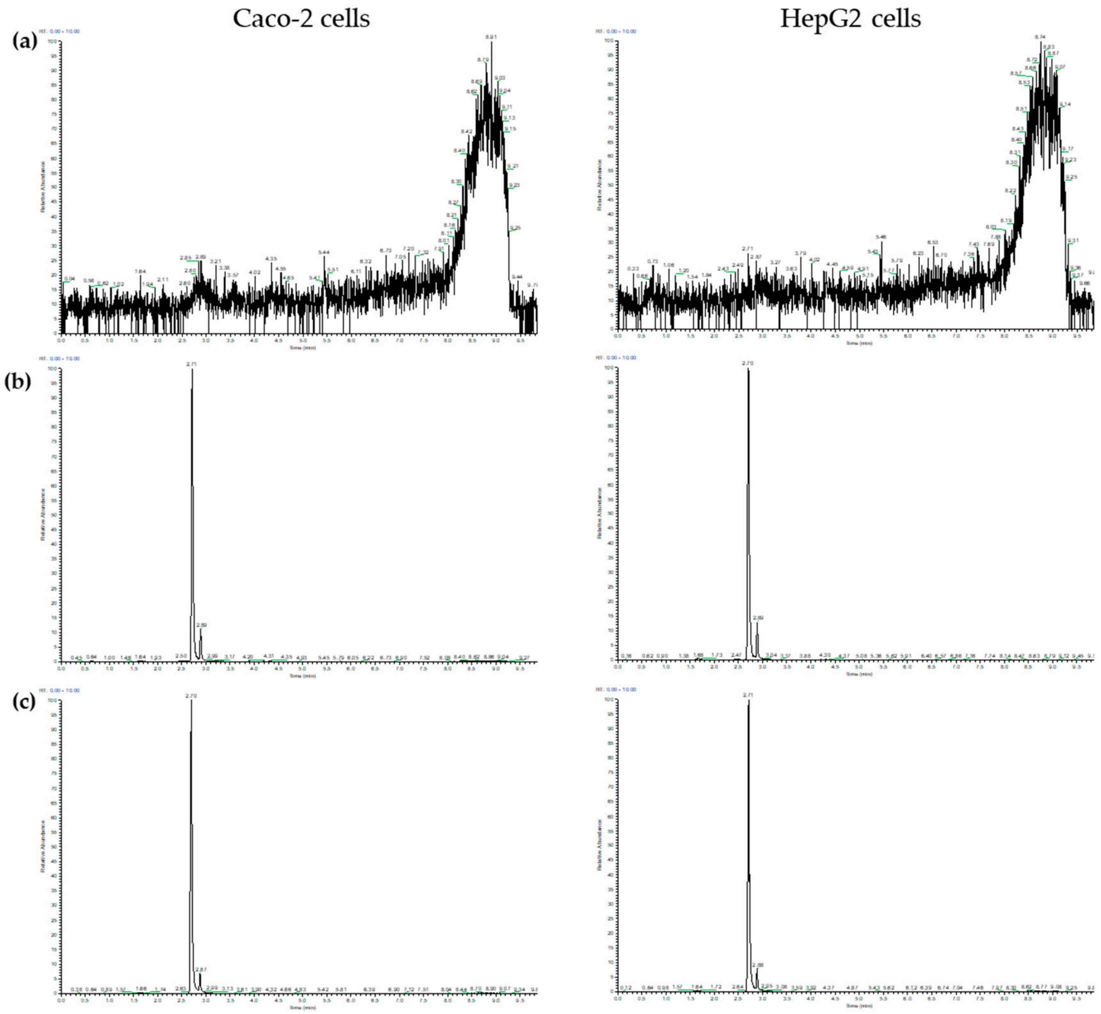


**Figure S2.** Chromatograms obtained by UHPLC-MS/MS of ATX-a in Caco-2 and HepG2 cells exposed to 50 μg/mL ATX-a fumarate for 24 h. (a) Negative control, (b) intracellular fraction (c) extracellular fraction.

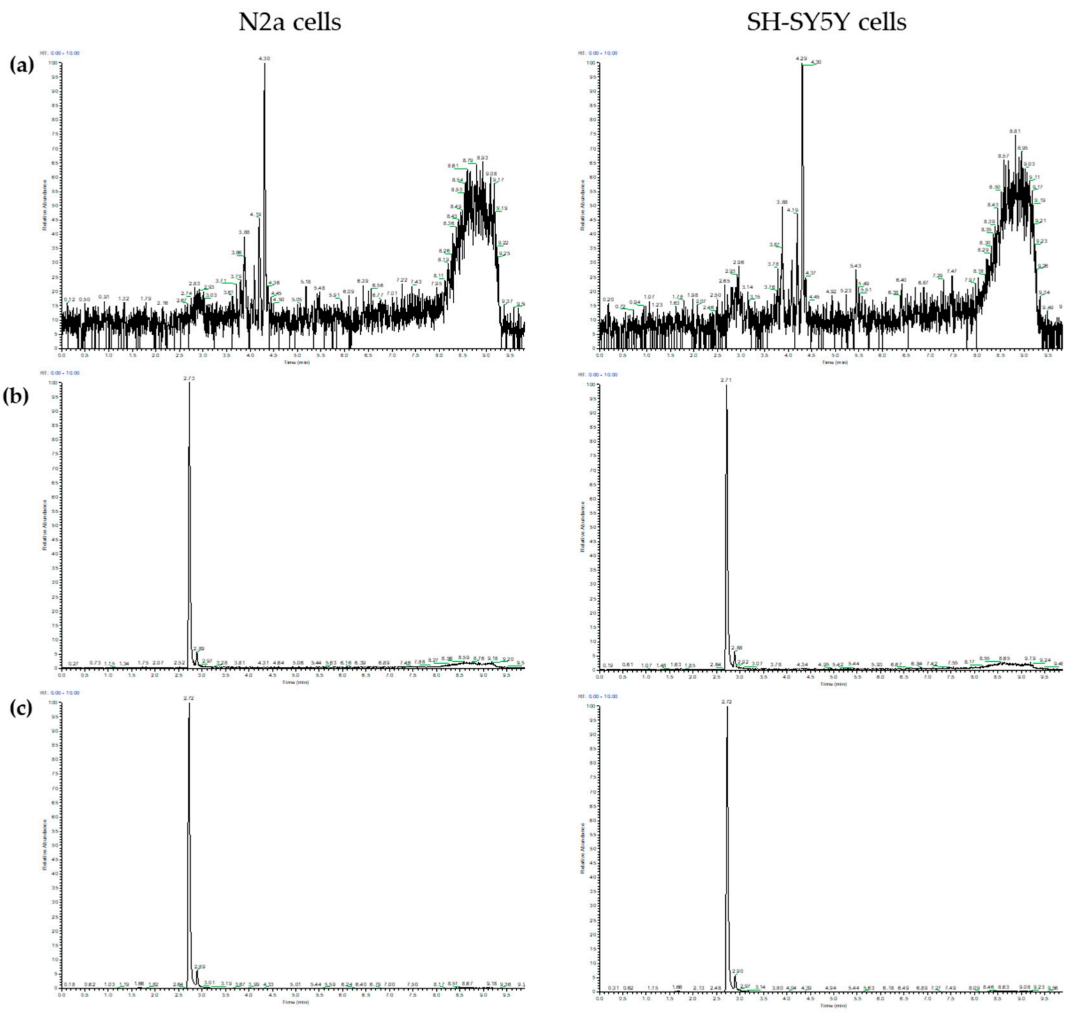


**Figure S3.** Chromatograms obtained by UHPLC-MS/MS of ATX-a in N2a and SH-SY5Y cells exposed to 50 µg/mL ATX-a fumarate for 24 h. (a) Negative control, (b) intracellular fraction (c) extracellular fraction.

**Table S1.** Primers used for PCR reactions in this study for responses to apoptosis (BAX, BCL-2), and necrosis (RIPK3) and the housekeeping gene (GAPDH).

| Mechanisms | Gene Symbol | Gene name | Reference |
|---|---|---|---|
| Apoptosis | BAX | BCL-2 associated X protein | qHsaCEP0040666 |
| | BCL2 | B-cell CLL/lymphoma 2 | qHsaCEP0058350 |
| Necrosis | RIPK3 | Receptor-interacting Serine/Threonine-Protein Kinase 3 | qHsaCEP0025866 |
| Housekeeping gene | GAPDH | Glyceraldehyde-3-phosphate dehydrogenase | qHsaCEP0041396 |
